# The Effects of Natural Products and Environmental Conditions on Antimicrobial Resistance

**DOI:** 10.3390/molecules26144277

**Published:** 2021-07-14

**Authors:** Lulu Huang, Saeed Ahmed, Yufeng Gu, Junhong Huang, Boyu An, Cuirong Wu, Yujie Zhou, Guyue Cheng

**Affiliations:** 1 MOA Laboratory for Risk Assessment of Quality and Safety of Livestock and Poultry Products, Huazhong Agricultural University, Wuhan 430070, China; huanglu@webmail.hzau.edu.cn (L.H.); guyufeng@webmail.hzau.deu.cn (Y.G.); 15827423532@163.com (J.H.); anboyu18327091764@163.com (B.A.); wcrvary@outlook.com (C.W.); 2 Department of Biological Sciences, National University of Medical Sciences, Rawalpindi 46000, Pakistan; saeed.ahmed@numspak.edu.pk; 3 College of Veterinary Medicine, Huazhong Agricultural University, Wuhan 430070, China; yujiezhou2021@163.com

**Keywords:** antimicrobial resistance, co-selection, antimicrobial peptides, heavy metal, microbial volatile compounds, stress factors

## Abstract

Due to the extensive application of antibiotics in medical and farming practices, the continued diversification and development of antimicrobial resistance (AMR) has attracted serious public concern. With the emergence of AMR and the failure to treat bacterial infections, it has led to an increased interest in searching for novel antibacterial substances such as natural antimicrobial substances, including microbial volatile compounds (MVCs), plant-derived compounds, and antimicrobial peptides. However, increasing observations have revealed that AMR is associated not only with the use of antibacterial substances but also with tolerance to heavy metals existing in nature and being used in agriculture practice. Additionally, bacteria respond to environmental stresses, e.g., nutrients, oxidative stress, envelope stress, by employing various adaptive strategies that contribute to the development of AMR and the survival of bacteria. Therefore, we need to elucidate thoroughly the factors and conditions affecting AMR to take comprehensive measures to control the development of AMR.

## 1. Introduction

Antimicrobial resistance (AMR) is a threatening global health problem, with an expected 10 million deaths per year by 2050 [1]. The overuse of antibiotics creates selective pressure contributing to the emergence of AMR (it is worth noting that antimicrobials include antibiotics, antivirals, antifungals, and antiparasitics and that AMR in this article refers specifically to the resistance of bacteria to antibiotics). Antibiotics are the secondary metabolites produced by a range of microorganisms. Due to the abuse of antibiotics on human and animal pathogens, the polluted environment accumulates antibiotic resistance genes (ARGs) and serves as a reservoir that can then transfer from the environment to humans and animals via mobile genetic elements (namely, horizontal gene transfer, including integrons, transposons, and plasmids) [2,3]. Nucleotide sequence homology between the multidrug-resistant soil bacteria and human pathogen has also been reported, which is a major reason for fast AMR dissemination [4]. Surprisingly, metagenomic and functional studies have identified that determinants of resistance existed in the primitive and ancient eras [5,6,7]. Although ARGs are naturally originated, they will only show the resistance to antibiotics under selection pressure [8].

Microbial- or plant-derived antimicrobial substances, host defense peptides from various organisms, and microbial volatile compounds that are usually used as antimicrobials can also develop selective resistance [9,10]. Bacteria exposed to non-antibiotic substances with antibacterial activity (antimicrobial metals and antibacterial biocides) may induce co-selection of resistance to antibiotics [11]. Furthermore, nutrients, temperature, pH, reactive oxygen and nitrogen species, etc. activate bacterial stress response and influence bacterial susceptibility to antimicrobials [12]. Here, we provide a brief overview of recent studies concerning the effects of natural products and environmental conditions on AMR.

## 2. Selection of Antimicrobial Resistance by Natural Products

### 2.1. Selection of AMR by Plant-Derived Antimicrobial Compounds

As shown in Table 1, many plants are abundant in bioactive secondary metabolites that have antimicrobial activity (phytochemicals). Plant-derived antimicrobial compounds exert their antimicrobial activity in several different ways, including disruption of the bacterial membrane, inhibition of cell wall or protein synthesis, damage to the synthesis and function of DNA/RNA, interference with intermediary metabolism, induction of coagulation of cytoplasmic constituents, and interruption of normal cell communication, such as alkaloids, flavonoids, quinones, tannins, coumarins, terpenes, lectins, and saponins [13,14]. Surprisingly, the antimicrobial properties of many plant-derived antimicrobial compounds (such as catechins, ferulic acid, and their derivatives) rely on their ability to perturb the redox balance of target cells and the antioxidant properties needed for antimicrobial activities [15,16].

There are numerous studies demonstrating that some plant extracts such as conessine (against multidrug-resistant *P. aeruginosa*), extracts from pomegranate peel, milk thistle seeds, and reserpine (inhibitor of multidrug efflux transporter STY4874) have the ability to modulate AMR, probably because they can restore the antibiotic susceptibility by inhibiting efflux pump systems [19,20]. Extracts from Pilgerodendron uviferum, 5’-methoxyhydnocarpina, a natural product from Hypericum olympicum, and Kaempferol Rhamnoside isolated from Persea Lingue Nees all have the inhibitory activity of NorA efflux pump of the *S. aureus* [21,22,23,24]. What is more, several plant extracts can inhibit the formation of bacterial biofilms that contributes to AMR; for instance, trans-cinnamaldehyde has an obvious effect on removing the biofilm of uropathogenic *E. coli* [25]. The essential oils of Cymbopogon citratus and Syzygium aromaticum and the components of lemongrass oil show the capacity to disrupt biofilms [13]. Additionally, plant-derived products have the ability to reduce the virulence of bacteria and regulate the host’s immune response, thereby affecting the survival of bacteria [13].

One way for plant-derived compounds to act as resistance modifying agents is the use of plant extracts combined with antibiotics, which is greater than the sum of each drug’s individual efficacy [26]. Using the combination of antibiotics and plant extracts reduces the minimum inhibitory concentration (MIC) value of antibiotics, thereby increasing the susceptibility of bacteria to drugs [26]. Kuok et al. studied the synergistic effect of the combination of antibiotics and herbal extracts on MRSA, and the results showed that the D. genkwa extract inhibited the synthesis of peptidoglycan in the bacterial cell wall by combining with PBPs, thus enhancing the effect of oxacillin, which will become a new strategy for combined treatment [27].

Bacteria are less likely to develop resistance to plant-derived antibacterial agents, as many antibiotics only involve a single target, while plant-derived antibacterial agents contain a variety of active ingredients that own various mechanisms of action against several targets [26]. However, studies on antimicrobial properties of herbal drugs on clinical isolates indicated that there were still some insusceptibility or resistance in microbes towards some naturally occurring antimicrobial compounds [28]. Khan et al. found that the multidrug-resistant (MDR) strains of *E. coli*, *Klebsiella pneumonia*, and *Candida albicans* show resistance to the herbal extracts of Terminalia arjuna and Eucalyptus globulus [29]. A multistep exposure of a wild-type *Salmonella enterica* serovar Senftenberg to sublethal concentrations of linalool, one of the components of plant extract basil oil, which is widely used in the food, perfume, sanitary, and cosmetic industries, not only induces resistance not only to linalool but also to several antibiotics including trimethoprim, sulfamethoxazole, piperacillin, chloramphenicol, and tetracycline [30]. Similar phenomena are also found in other strains, suggesting that the resistance to plant-derived extracts may select cross-resistance to antibiotics in several pathogens, which may endanger public health [30]. Therefore, we must fully understand the possible effects of plant extracts on bacteria. At present, there are still very few research studies on the mechanisms of resistance to herbs. One study has shown that deletion of the *sigB* gene in *Listeria monocytogenes* decreased the resistance to carvacrol; however, research on herb drug resistance genes still needs to be more in-depth [31].

### 2.2. Selection of AMR by Antimicrobial Peptides

Antimicrobial peptides (AMPs), also known as host defense peptides, are present in almost every form of life, such as bacteria, plants, insects, and viruses [32]. The antimicrobial mechanism of AMPs includes the ability to change membrane permeability and to interact with a series of intracellular target molecules [33]. Different from traditional antibiotics, it is generally believed that AMPs preferentially attack cell membranes so that they will not cause extensive AMR, but their ability to recognize specific targets still provides possibilities for gene mutations and antibiotic resistance [34]. AMPs are likely to be substituted for antibiotics in the future due to their broad-spectrum antibacterial activity against Gram-negative and Gram-positive bacteria, fungi, parasites, viruses, and tumor cells, as well as their lower likelihood of inducing AMR [33].

Nevertheless, studies have confirmed that certain bacteria are inherently resistant to AMPs due to the lack of electrostatic attraction between the bacterial cell membrane and AMPs [33,35]. Additionally, bacteria develop resistance to AMPs by using a variety of proteases secreted by bacteria and extracellular structures or by increasing efflux [36]. Meanwhile, bacteria can adaptively modify cell surface structures, thus hindering AMPs from electrostatic adsorption with bacteria [36]. Moreover, AMP-binding proteins produced by bacteria prevent AMPs from contacting cells by binding to AMPs. The typical representative of this protein is staphylokinase produced by *S. aureus*, which can resist human defensins [37]. In Gram-positive bacteria, the negative charge on the cell wall surface is reduced due to the combination of some positively charged substances and the cell wall teichoic acid, which leads to the formation of resistance to cationic AMPs. For example, *S. aureus* induces AMPs resistance by activating the D-alanylation of TA, incorporating lysylphosphatidylglycerol into the bacterial membrane and activating vraFG AMP transporter [34].

### 2.3. Selection of AMR by Microbial Volatile Compounds (MVCs)

Bacteria and fungi release a series of organic and inorganic volatile compounds such as alkanes, alkenes, alcohols, esters, ketones, terpenoids, sulfur-containing compounds, and a small group of inorganic compounds that act as antimicrobials, antifungal agents, or the modulators of AMR [9]. Some of the specific volatiles generated by a few microorganisms have been indicated as potential antimicrobial agents, as shown in Table 2. Examples include the hormone-like g-butyrolactones with broad-spectrum activity against bacteria, fungi, and yeast; furfuryl isovalerate that inhibit the growth of Gram-positive and Gram-negative bacteria; while volatiles produced by actinomycetes inhibit the growth of *Bacillus subtilis* [9]. MVCs combined with antibiotics can also enhance their antibacterial efficacy, as terpenes have a synergistic effect in a complex with penicillin [9,38].

It has also been reported that MVCs can enhance the resistance of microorganisms by modifying membrane permeability [39,40], inducing of efflux pumps [41], stimulating the formation of persisters [42,43], and alleviating the oxidative stress imposed by antibiotics [44] (Table 2).

**Table 2:** Modulation of AMR by microbial volatile compounds (MVCs).

MVCs	Origin Microorganism	Target Microorganism	Antimicrobial Resistance	Mechanism	References
Trimethylamine	*E. coli*	*E. coli*, *P. aeruginosa*, *S. aureus*, and *B. subtilis*	Tetracycline	Increasing the transmembrane pH and lowering the transport of tetracycline inside the cell	[39]
Ammonia (NH_3_)	*E. coli*	*E. coli*	Tetracycline	Promoting intracellular accumulation of polyamines by modifying membrane permeability	[40]
Indole	*E. coli*	*Pseudomonas putida*	ampicillin	Inducing the *Pseudomonas* TtgGHI antibiotic efflux pump	[41]
2,3-Butanedione and glyoxylic acid	*B. subtilis*	*E. coli*	Ampicillin and tetracycline	The induction of the expression of *hipA* and *hipB*, TA system related genes, resulting in bacterial persistence	[42]
2-Aminoacetophenone	*P. aeruginosa*	*Acinetobacter baumanii*	Meropenem and tetracycline	Stimulating persisters formation	[43]
Hydrogen sulfide (H_2_S)	*Bacillus anthracis*, *P. aeruginosa*, *S. aureus*, and *E. coli*	*B. anthracis*, *P. aeruginosa*, *S. aureus*, and *E. coli*	A range of different antibiotics targeting DNA, RNA, cell wall, or protein biosynthesis	Mitigation of oxidative stress imposed by antibiotics	[44]
Dimethyl trisulfide, 1-methylthio-3-pentanone and o-aminoacetophenone	*Burkholderia ambifaria*	*E. coli*	Aminoglycosides, such as gentamicin and kanamycin	Unknown	[45]
Nitric oxide (NO)	Many Gram-positive bacteria, such as *B.subtilis* and *S. aureus*	NO producing and non-producing microorganisms	A broad spectrum of antibiotics such as cefuroxime	Chemical modification of toxic compounds and the alleviation of the oxidative stress	[46]

### 2.4. Selection of AMR by Antibiotics, Biocides and Heavy Metals

Biocides (e.g., disinfectants, antiseptics, and preservatives) are widely used in farms and slaughterhouses [47]. In animal husbandry, heavy metals are added in animal feed as growth promoters (e.g., copper sulfate and zinc oxide) [11], nutritional trace minerals (e.g., Zn, Cu, chromium (Cr), vanadium (V), tin (Sn), nickel (Ni), and molybdenum (Mo)), or antimicrobials [48]. Bacteria evolve resistance to antibiotics under the selective pressure, while biocides and heavy metals also have the ability to co-select for antibiotic-resistant bacteria [49]. Heavy metal contamination of source water may contribute to the development, co-selection, and dissemination of bacterial AMR [50]. By using a copper shock loading test, Zhang et al found that resistance to the antibiotics tested was significantly increased, and the relative abundance of most detected ARGs (especially MGEs int I and transposons) was higher [50]. Surprisingly, even in the absence of antibiotics and heavy metals, resistance can still exist for a period of time [50].

Co-selection of biocides/metal and antibiotic resistance is mainly achieved by co-resistance (resistance genes to both antibiotics and biocides/metals are co-located in the same cell) or cross-resistance mechanisms (e.g., over-expression of multi-drug efflux pumps, which can expel a wide range of substrates such as antibiotics, biocides, and heavy metals) (Table 3) [49,51]. The use of feeds containing high levels of zinc in commercial swine herds may select MRSA because the zinc resistance gene (*czrC*) and the methicillin resistance gene (*mecA*) are co-located in the staphylococcal cassette chromosome mec (SCCmec) [52,53,54]. *tcrB*, a new Cu resistance gene, and the genes that confer resistance to macrolide and vancomycin are on the same conjugated plasmid; therefore, the use of Cu is associated with the co-selection of macrolide and vancomycin resistance for *enterococci* [55,56,57]. If antibiotic resistance genes (ARGs) and the biocide/metal resistance genes (BMRGs) are co-occurring in plasmids or other genetic elements (i.e., integrons, transposons), it poses risk to the spread of resistance across the environment and animal and human populations. An example is class 1 integrons, which encode a quaternary ammonium compounds (QACs) efflux (*qacEΔ1*) plus sulphonamide resistance (*sul1*) and other ARGs [11]. Another example is the class 1 integron-integrase gene, *intI1*, which is related to zinc and cefotaxime resistance [58]. A novel Tn916-like element, Tn6087, confers resistance towards antibiotics and antiseptics that are encoded by *qrg*, a small multidrug resistance gene [59]. Genomic analysis indicated that the plasmid detected in *S. Infantis* isolate contained not only a class 1 integron, which harbored AMR gene cassette including integrase (*intI1*), aminoglycoside-phosphotransferase, and β-lactamase (*blaTEM–1*), but also two separate transposons, both of which harbored arsenic resistance operons [60]. Mbanga et al. found that co-resistance to heavy metal/biocide antibiotics were evident in multidrug-resistant *E. coli* isolates from wastewater sources and that the plasmids in these isolates contained the *chrA* (confer chromate resistance) gene and *qacEΔ1* gene (a disinfectant resistance gene) and the class 1 integron (including ARG cassette) [61].

Cross-resistance to antibiotics and biocides/metals is mainly mediated by enhanced efflux pump activity or gene mutations. In *Enterobacteriaceae*, cross-resistance is potentially possible via AcrAB-tolC pumps, a member of resistance-nodulation-division (RND) family, while pumps homologous to AcrAB-TolC exist in other species of Gram-negative bacteria, for instance, MexAB-OprM, MexCD-OprJ, and MexXY-OprM in *P. aeruginosa* and CmeABC in *Campylobacter jejuni* [76]. A multi-drug efflux pump encoded by the chromosomal gene *norA* in *S. aureus* has been shown to expel norfloxacin, ciprofloxacin, benzalkonium chloride, and chlorhexidine [77]. Similarly, chromosomal gene *mdrL* that encodes multi-drug efflux pump in *L. monocytogenes* confers resistance to macrolides, cefotaxime, and heavy metals [69]. Table 4 lists selected studies that have reported the multi-drug efflux pumps that confer cross-resistance to antibiotics and biocides/metals. If expression of resistance systems to metals and antibiotics are controlled by a common regulator, the changes of the regulator will co-regulate antibiotics and metals resistance. For example, TCS CzcRS controls the expression of not only the czcCBA efflux pump which expel cadmium, zinc and cobalt, but also the OprD porin, conferring resistance to carbapenems [12]. 

## 3. Selection of AMR by Other Environmental Stress Factors

Bacteria always come across with numerous pressures in their usual surroundings [12]. The exposure to, e.g., oxidative stress, nutrient stress, heat shock stress, ribosomal stress, and envelope stress all influence the bacterial susceptibility to a range of antibiotics [12,86]. How these environmental pressures affect AMR is described below (Table 5).

### 3.1. Effects of Nutrients on AMR

Nutrient starvation/limitation (e.g., amino acid deprivation, carbon sources or fatty acids, depletion of Fe, phosphate) activates a stringent response by inducing the *relA* and *spoT* genes to synthesize the alarmonguanosine 5′-(tri)diphosphate 3′-diphosphate (ppGpp), which has a myriad of effects on bacterial physiology and antimicrobial susceptibility, such as by inhibiting the peptidoglycan biosynthesis that leads to a reduced susceptibility of *E. coli* to penicillin [87]. Additionally, the activated stringent response also promotes AMR by decreasing the synthesis of 4-hydroxy-2-alkylquinolines (HAQ) and increasing antioxidant defenses [12]. Nguyen et al. found that the increases in HAQ levels enhanced the antibiotic susceptibility and also increased hydroxyl radical production levels in ΔrelAΔspoT mutant of *P. aeruginosa* biofilm, suggesting that the growth-arrested, nutrient-limited, metabolically slowed cells are likely to resist the attack of severe different types of antibiotics in this way [88].

In contrast, high concentrations of exogenous metabolites may turn the antibiotic-resistant phenotype into an antibiotic-sensitive one by affecting the metabolomics of the bacteria. This is a new method for controlling the generation of AMR. This phenotypic shift is the reprogramming of the state of the metabolome by exogenous metabolites (such as glucose, fructose, etc.) to a state that facilitates the uptake of antibiotics by bacteria [89]. For example, the sugars associated with the glucose metabolism pathway (e.g., glucose, mannose, fructose) can enter the glycolytic pathway to produce nicotinamide adenine dinucleotide ( NADH ), which then produces more proton-motive force (PMF) by the electron transport chain. Allison et al. have shown that by adding these metabolically related sugars, the increasing in PMF enhances the ability of persister to uptake more aminoglycosides, thereby the drug regains its effect [90]. Additionally, exogenous glucose, combined with a sub-lethal dose of antibiotics, can interfere with various metabolic pathways of MRSA and methicillin-susceptible *S. aureus*, accordingly enhancing the bactericidal effect of antibiotics [91]. Studies have validated that changing the bacterial metabolism from a quiescent to a replicating state upsurges the susceptibility to antimicrobial compounds. Allison proved that upon the addition of the metabolites, mannitol, or fructose, persisters became hypersensitive to aminoglycosides [90]. Mannitol and fructose stimulated central carbon metabolism by inducing the PMF in NGMA (non-growing but metabolically active) cells, thereby enabling aminoglycoside uptake and consequent killing [92].

Similarly, in amino acid metabolism, studies using proteomic analysis have shown that glutamine degradation, aspartate, and asparagine metabolic pathways are overexpressed in the outer membrane proteome of *Klebsiella pneumoniae* resistant to colistin [93]. Studies have shown that exogenously added alanine, converted to acetyl-CoA through metabolic pathways, enters the Krebs cycle, then enhances the uptake of kanamycin by increasing NADH production and proton-dynamic PMF and restores *Edwardsiella tarda* susceptibility to antibiotics [94]. Similar results were observed in other Gram-negative bacteria (*Vibrio parahaemolyticus*, *K. pneumoniae*, *P. aeruginosa*) and Gram-positive bacteria (*S. aureus*), which confirmed the relationship between bacterial metabolism and AMR [94].

### 3.2. Selection of AMR by Oxidative Stress

Reactive oxygen species (ROSs) (e.g., reactive oxygen and nitrogen species) destroy a range of cellular macromolecules and thus provoke adaptive oxidative stress responses in bacteria predisposed to survival in the presence of this stressor. Interestingly, some bactericidal antibiotics (e.g., aminoglycosides, fluoroquinolones, and beta-lactam antibiotics) induce the formation of endogenous ROSs in bacteria, which also lead to oxidative stress [95]. The antioxidant mechanisms of bacteria cells, including both enzymatic and non-enzymatic antioxidant systems, are recruited in response to oxidative stress. Enzymatic antioxidant systems play a major role. Related enzymes mainly include superoxide dismutase (decomposing superoxide to form H_2_O_2_, which is subsequently further degraded by catalytic enzymes) and glutathione peroxidase (GPX) (degrading H_2_O_2_ and organic hydroperoxide). In addition, some other enzymes, such as hyaluronidase and peroxidase and small redox protein (thioredoxin), are also considered as antioxidants that can counteract the damage caused by oxidative stress [96]. Bacteria can also produce enzymes that repair oxidative damage, including enzymes involved in DNA repair, as well as proteolytic enzymes and lipolytic enzymes.

An effective strategy against antimicrobial-resistant bacteria is to destroy the antioxidant mechanisms of bacterial pathogens. For example, ebselen, a bacterial thioredoxin reductase inhibitor, blocks the antioxidant defenses of multidrug-resistant Gram-negative bacteria, which possess both thioredoxin and glutathione systems when combined with silver [97]. Additionally, the combination of two ROS-generating antimicrobial compounds exhibited a strong synergetic effect against *Rhodococcus equi* [98]. Novel antibacterial substances that produce ROS are also attracting more and more attention. One of them is AGXX^®^—which is composed of two transition metals, silver and ruthenium—causes an oxidative and metal stress response as well as strong protein damage to kill MRSA [16]. Similarly, degradable Cu-doped phosphate-based glass (Cu-PBG) nanozyme has an effective antibacterial activity against Gram-positive and Gram-negative bacteria both in vitro and in vivo, by increasing the level of ROSs and releasing copper [99].

The expression of numerous multidrug efflux pump systems was positively influenced by causes of oxidative stress [12]. One of the redox-responsive regulators of multidrug efflux is SoxRS (response to, e.g., superoxide) regulating multidrug efflux AcrAB-TolC and porin OmpF [100]. The SoxRS system mediates the expression of *micF* and *acrAB* genes, while the increased expression of *micF* leads to a decrease in outer membrane porin OmpF and a decrease in cell permeability, and the *acrAB* genes encodes a multidrug efflux pump [100]. The two modes of action jointly participate in the resistance of *E. coli* and *Salmonella* mediated by the SOxRS system [101]. MexR (response to, e.g., peroxide or cumene hydroperoxide) suppresses the MexAB-OprM efflux pump operon. *P. aeruginosa* perceives the peroxide stimulation to cause the dissociation of MexR from the target MexAB-OprM promoter DNA, and the expression of efflux pump genes increases, which induces resistance of *P. aeruginosa* [102]. MgrA (response to, e.g., peroxide and organic hydroperoxide), a homolog of the MarR family of multiple-antibiotic-resistance proteins, negatively regulate effluxes NorA, NorB, and Tet38, which confer resistance to quinolone-type antibiotics such as ciprofloxacin and norfloxacin in *S. aureus* [103]. The PA5471 gene (response to ROS) regulates MexXY-OprM, which promotes aminoglycoside resistance development in *P. aeruginosa* [104].

### 3.3. Effect of Temperature, pH, and Osmotic Pressure on AMR

Recently, a study analyzed the antibiotic consumption and AMR globally against several latent contributing factors [105]. It was found that among the measures of climate, the average temperature was strongly and positively correlated with AMR indices [105]. However, in some of the particular settings, the situation is different. For example, in wastewater lagoons at cattle feedlots, the level of ARGs in the autumn was 10~100 times greater than that in summer [106]. In effluents of the wastewater treatment plant, higher release rates of ARGs were observed in winter than in spring [107]. The higher quantity of ARGs in winter may be linked with the increasing selective pressure caused by antibiotics that were used to control the disease as well as higher antibiotic loading rates as pollutants [108]. Thus, the temperature was not always the sole and absolute factor determining ARGs.

Sometimes, extraordinary temperature appears to portend the eradication of ARGs. It was demonstrated that the abundance of ARGs in the swine manure composting process could be reduced due to the increasing of temperature [109]. During anaerobic digestion (AD) processes, the temperature was considered as one of the most dominant factors for removing ARGs and decreasing the HGT [110,111,112]. Since the abundance profiles of particular ARG and metal resistant gene (MRG) types might correlate with characteristics of specific microbial communities, the much narrower ecology of microorganisms under higher temperatures may lead to the improvement of removal of ARGs [113]. In other cases, high temperature does not seem good for the elimination of ARGs. In swine wastewater, the abundance of ARGs was effectively reduced, but the removal efficiencies of ARGs was higher in winter than in summer [114]. In an integrated surface flow constructed wetland, 77.8% and 59.5% removal rates of total targeted ARGs were achieved in the winter and summer, respectively [115]. The mechanism and consistency of the conclusion for the effect of temperature on the elimination of ARGs still need further investigation.

A recent study found that cefotaxime- and ciprofloxacin-induced resistant strains exhibit similar or better survival rates than wild-type strains at sublethal temperature, pH, and osmotic pressure [116]. The result indicates that the resistant strains have the ability to regulate the effects of environmental stress by themselves. Similarly, *S. aureus*-resistant strains are more tolerant than sensitive strains after the exposure to different environmental stresses [117]. The effects of temperature, pH, and osmotic pressure on AMR are often related to the enveloping stress, regulated by TCSs or sigma factors [12]. For example, the RcsCDB/F phosphorelay system that plays a key role in the regulation of biofilm formation and pathogenicity in *Enterobacteriaceae* is activated by envelope stress, including low temperature, high osmolarity, and desiccation, and leads to β-lactam resistance in *E. coli* and polymyxin B resistance in *S. enterica* [118]. CpxRA (responses to alkaline pH and high osmolarity) modulates the integrity of the cell envelope in part by controlling peptidoglycan amidase activity, which causes *Salmonella* and *E. coli* to become resistant to CAPs [119]. Β-lactams resistance is due to the expression of multidrug exporter genes regulated downstream by CpxRA in *E. coli* [120]. Moreover, CpxR was found to play an important role in mediating oxidative stress, osmotic stress, and alkaline pH stress tolerance, as well as macrolide resistance in *Haemophilus Parasuis* [121]. EnvZ/OmpR senses osmotic pressure to form β-lactams resistance by the change of outer membrane porin proteins in *E. coli* and *S. enterica* [12]. pmrAB is also activated by low pH and low environmental Fe3+, independent of PhoPQ and low Mg^2+^, providing lipopolysaccharide modifications that promote *Salmonella* survival and CAPs resistance and virulence [122].

With respect to sigma factors that regulate the adaptive response of bacteria to environmental stress, sigma factor (σB) mediates rifampicin resistance in *B. subtilis* when exposed to high salt, heat, ethanol, low temperature, and acid pH [123] and is also involved in β-lactams, CAPs, glycopeptides resistance in *S. aureus* [124,125]. In *B. subtilis*, σM (response to high salt), σW (response to alkaline shock), and σX (response to high temperature) lead to bacitracin and ampicillin resistance [126]. Furthermore, rpoS encodes the general stress response sigma factor σS, which is a response to nutrient starvation, high osmotic pressure, acid pH, oxidative stress, and non-optimal extreme temperature, which is linked to AMR [127]. Following the RpoS-mediated general stress response and the σE-dependent envelope stress response, bacteria activate a stress-induced increase in the mutation rates under the control of the SOS response [128,129,130]. RpoS has also been implicated in low-temperature-promoted biofilm development in *E. coli* [131]. Other mechanisms include, e.g., the phosphoregulation of MgrA at low pH, which reverses its repression of *norB* expression, encoding the NorB multidrug resistance efflux pump and then causes *S. aureus* to evade the lethality of norfloxacin [132].

**Table 5:** Stress-inducible antimicrobial resistance mechanisms.

Stress	Resistance Mechanism	Stress-Responsive Regulator	Organism	References
Nutrient starvation/limitation	ppGpp-mediated inhibition of the peptidoglycan biosynthesis	Has not been identified	*E. coli*	[87]
	Decrease the synthesis of HAQ and increase antioxidant defenses	Has not been identified	*P. aeruginosa*	[12]
Oxidative stress	Multidrug efflux AcrAB-TolC and porin OmpF	SoxRS	*E. coli*	[100]
	Single point mutations in the *soxR* gene and elevated *soxS* expression	SOxRS	*E. coli* and *Salmonella*	[101]
	MexAB-OprM efflux pump	MexR	*P. aeruginosa*	[102]
	effluxes NorA, NorB, and Tet38	MgrA	*S. aureus*	[103]
	Multidrug efflux MexXY-OprM	PA5471 gene	*P. aeruginosa*	[104]
Enveloping stress	Biofilm formation and pathogenicity	RcsCDB/F phosphorelay system	*E. coli* and *S. enterica*	[118]
	Control peptidoglycan amidase activity	CpxRA	*E. coli*	[119,120]
	Outer membrane porin proteins	EnvZ/OmpR	*E. coli* and *S. enterica*	[12]
	Lipopolysaccharide modification	pmrAB	*Salmonella*	[122]
		sigma factors (σB, σM, σW, σX)	*B. subtilis*, *S. aureus* and *E. coli*	[123,124,125,126]

## 4. Discussion

The ecosystem is continuously exposed to several antimicrobial agents through wastewater, agricultural runoff, and animal-related and human activities. When bacteria face the various selective pressures encountered in the environment, they will trigger an adaptive response and protect the bacteria from the external stress and the attack of antibiotics. Apart from this, with the widespread use of antibacterial agents in the fields of agriculture and veterinary and human medicine, AMR has gradually increased, and ARGs have also been continuously enriched [133]. The spread of carriers in the environment that harbor ARGs but cannot infect the animal or human body would upsurge the diversity and abundance of ARGs in vectors that can settle and occasionally attack the animal or human body. Even without the use of antibacterial agents, certain heavy metals and other compounds with antibacterial activity (such as biocides) can possibly maintain or even increase the resistance of bacteria to antibiotics through co-selection [11]. When bacteria are exposed to environments contaminated with heavy metals are or where disinfectants are often used, the co-occurrence of resistance genes to antibiotics, biocides, and metals on mobile genetic elements can be attributed to the dissemination of antibiotic resistance from environment to animals and human populations via food chains. In addition to heavy metals, natural bacterial metabolites, microbial volatile compounds, etc. all affect the resistance of bacteria to varying degrees, and the mechanisms that affect bacteria’s susceptibility are similar to those that affect resistance to antibiotics.

The “Golden Age” of antibiotics saw the development of hundreds of antimicrobials for curing infectious diseases. However, this era no longer exists. The agri-food industry is under stress to decrease the use of antibiotics. Considering the human health risk due to emerging of AMR in foodborne pathogens, some of the novel ecofriendly de-contamination and sanitation strategies that maintain a high level of antimicrobial activity are used in some food-processing facilities to reduce the presence of the foodborne pathogens. However, according to the latest research progress, the exposure of *Salmonella* spp., *E. coli*, and *L. monocytogenes* to the novel but not yet widely used nonthermal microbial decontamination, UV light, and nonthermal atmospheric plasma can select variants with increased resistance to several clinically relevant antibiotics, which can contribute to the spread of AMR along the food chain [134]. On the other hand, many countries have banned the use of antimicrobials as animal growth promoters and the application of antimicrobials critically important in human clinical treatment in the veterinary field. However, the effort to reduce the application of antimicrobials in agri-food production alone will not yield the required outcome in terms of limiting consumer exposure, since a range of pollutants are excreted with animal waste, including veterinary antimicrobials, heavy metals nutrients, and pathogens, which can enter local farmland soils, surface water, and groundwater and which pose direct or indirect human health risks. Additionally, the use of veterinary antibiotics and feed additives in the veterinary field should be measured, which not only will make food of animal origin less harmless to consumers but also will render the manure more benign for treatment and removal on farmlands. In addition, humans need to monitor the use of antimicrobials/antibiotics in communities, animals, and hospitals, and they need to monitor residual amounts, resistant microorganisms/bacteria, and resistant genes in all parts of the environment and continuously update the information. The crisis of antimicrobial/antibiotic resistance has reached an uncontrollable rate, and if immediate measures are not taken to solve the problem, simple microbial infections may lead to life-threatening ones [135].

## Figures and Tables

**Table 1:** The mechanisms of the secondary metabolites with potential antimicrobial properties.

Category	Plant-Derived Compounds	Antimicrobial Effects	Mechanism	References
Flavonoids	Aspilia mossambicensis, Ocimum gratissimum, and Toddalia asiatica extracts	Methicillin-resistant *Staphylococcus aureus* (MRSA) and *P. aeruginosa*	The interaction with membrane proteins resulted in increased cell membrane permeability and the disruption of the cell wall	[13]
Alkaloids	Berberine presented in roots and stem-bark of Berberis species	Antibacterial, antifungal, antiviral effects	Insertion of DNA to RNA polymerase, gyrase and topoisomerase IV, and nucleic acid	[17]
	The ethanol extract of Tabernaemontana catharinensis root bark	*Staphylococcus aureus*, *Escherichia coli*, and *Pseudomonas aeruginosa*	Endogenous indole that plays a role	
Terpenes	Sesquiterpenes isolated from different plants	The inhibition of the growth of Gram-positive bacteria and *Mycobacterium tuberculosis*	Not fully understood	[17]
Quinones	2,6-dimethoxy-1,4-benzoquinone (DMBQ) extracted from wheat germ	*S. aureus* and *Bacillus cereus*	Provide free radicals to irreversibly bind to the nucleophilic amino acids in microbial protein to cause protein function loss	[18]

**Table 3:** Mechanisms of co-selection of AMR by heavy metals.

Mechanism	Microorganism	Example	Reference
Co-resistance	MRSA	Plasmids carrying resistance genes for Cu and Cd (*copA*, *mco*, and *cadDX*) and multiple antimicrobials including macrolides, lincosamides, streptogramin B, tetracyclines, aminoglycosides, and trimethoprim (*erm(T), tet(L), aadD*, and *dfrK*)	[62]
	MRSA	Physical presence of the Zn resistance gene (*czrC*)on the methicillin resistance-encoding SCCmec element	[63,64]
	*Pseudomonas* sp.	A number of ARGs such as *aadA2*, *qacEΔ1*, and sulI (resistance against streptomycin, spectinomycin, quaternary ammonium, and sulfonamide) located in Tn5045, where chromate resistance genes *chrBACF* are obtained	[65]
	monophasic *S. Typhimurium*	Heavy metal resistance genes ((silver/copper (*silA*-*silE*), mercury (*merA*)) and ARGs exist on chromosomes or plasmids in *S. Typhimurium* strains from different sources	[66]
	*Enterococcus faecium* and *Enterococcus faecalis*	Co-transfer of *tcrB* and *erm(B)* genes between *E. faecium* and *E. faecalis* strains	[67]
	*E. faecalis*	The antibiotic resistance gene *tetM* (resistance to tetracycline), *vanA* (encoding vancomycin resistance), streptothricin acetyltransferase gene, and aminoglycoside adenylyltransferase gene was identified in Cu-resistant *E. faecalis*	[68]
Cross-resistance	*L. monocytogenes*	A multidrug efflux pump MdrL found in *L. monocytogenes* confers a resistant phenotype against a range of antimicrobial compounds and heavy metals, such as Zn, Co, and Cr	[69]
	*E. coli*	The membrane stress-responsive two-component system (TCS) CpxRA that is linked to resistance to a variety of cell envelope-targeting drugs in Gram-negative bacteria, is also Cu-responsive and contributes to Cu tolerance	[70]
	*P. aeruginosa*	TCS CscRS found in *P. aeruginosa* not only influences the transcription of the czcCBA operon that encodes an RND-type efflux pump, which confers resistance to Zn, Cd, and cobalt (Co), but also reduces the expression of a specific porin OprD through which imipenem enters the bacterium	[71]
	*E. coli*	The envelope stress response sigma factor RpoE activated by polymyxin B and linked to polymyxin B resistance in a number of Gram-negative bacteria is also activated by Zn in *E. coli* and contributes to Zn and Cu tolerance in *E. coli*	[72]
	*E. coli*	Exposure to Cu increases expression of the oxidative stress-responsive soxS regulatory gene that has been linked to expression of the acrAB multidrug efflux operon	[73]
Biofilm formation	*S. aureus*	The biofilm matrix enables bacteria to survive under stress conditions, such as exposure to heavy metals, and this in turn drives the frequency of mutation in the bacterial genomes, some of which may co-select for AMR	[74]
Facilitation of HGT	*E. coli*	Sub-inhibitory concentrations of heavy metals accelerate the horizontal transfer of plasmid-mediated ARGs in water environment by promoting conjugative transfer of ARGs between *E. coli* strains	[75]

**Table 4:** A list of multi-drug efflux pumps that confer cross-resistance to antibiotics, biocides/metals.

Efflux Pump Family	Multi-Drug Efflux Pump	Microorganism	Cross-Resistance	References
Resistance-nodulation-division (RND) family	MexAB-OprM	*P. aeruginosa*	Fluoroquinolones, biocides such as benzalkonium chloride	[78]
	MexCD-OprJ	*P. aeruginosa*	Ciprofloxacin and triclosan	[79]
		*P. aeruginosa*	Polymyxin B and benzalkonium chlorides	[80]
	AcrAB-tolC	*Enterobacteriaceae*	A wide range of antibiotics, dyes, detergents, and disinfectants	[81]
	AcrEF-TolC	*Salmonella enterica* serovar *Typhimurium*	A series of biocides such as Virkon, Superkill, AQAS, and Trigene, as well as reduced susceptibility to multiple antibiotics including nalidixic acid, chloramphenicol, tetracycline, and ciprofloxacin	[82]
	cmeABC	*C. jejuni*	A range of antibiotics, heavy metals, bile salts, and other antimicrobial agents	[83]
Plasmid-mediated quinolone resistance (PMQR) family	oqxAB	Mainly *Enterobacteriaceae* such as *E. coli* and *Salmonella*	Quinoxalines, quinolones tigecycline, nitrofurantoin, several detergents and disinfectants (benzalkonium chloride, triclosan, and SDS)	[84]
	SmeDEF	*Stenotrophomonas maltophilia*	Quinolones and triclosan	[85]
	Chromosomal gene *mdrL* that encodes multi-drug efflux pump	*L. monocytogenes*	Macrolides, cefotaxime and heavy metals	[69]
	the chromosomal gene *norA* that encodes multi-drug efflux pump	*S. aureus*	Norfloxacin, ciprofloxacin, benzalkonium chloride, and chlorhexidine	[77]

## Data Availability

Not applicable.

## Abbreviations

AD	anaerobic digestion
Ag	silver
AMR	antimicrobial resistance
AMPs	antimicrobial peptides
ARB	antimicrobial-resistant bacteria
ARG	antimicrobial-resistant gene
As	arsenic
CAP	cationic antimicrobial peptide
Cd	cadmium
Co	cobalt
Cr	chromium
Cu	copper
ESBL	extended-spectrum β-lactamase
Hg	mercury
HGT	horizontal gene transfer
MGE	mobile genetic element
Mo	molybdenum
MRSA	methicillin-resistant *Staphylococcus aureus*
MRG	metal resistant gene
MVC	microbial volatile compound
Ni	nickel
Pb	lead
ROS	reactive oxygen species
Sn	tin
SCV	small-colony variants
TCS	two-component system
V	vanadium
Zn	zinc
